# Radioiodinated indomethacin amide for molecular imaging of cyclooxygenase-2 expressing tumors

**DOI:** 10.18632/oncotarget.15437

**Published:** 2017-02-17

**Authors:** Agnieszka Morgenroth, Andreas T.J. Vogg, Bernd Neumaier, Felix M. Mottaghy, Boris D. Zlatopolskiy

**Affiliations:** ^1^ Department for Nuclear Medicine, RWTH Aachen University Hospital, 52074, Aachen, Germany; ^2^ Institute of Radiochemistry and Experimental Molecular Imaging, University Clinic Cologne, 50937, Cologne, Germany; ^3^ Max Planck Institute of Metabolism Research, 50931, Cologne, Germany; ^4^ Institute of Neuroscience and Medicine, Nuclear Chemistry (INM-5), Research Center Jülich, 52425, Jülich, Germany; ^5^ Department of Nuclear Medicine, Maastricht University Medical Center, 6229 HX, Maastricht, The Netherlands

**Keywords:** cyclooxygenase-2, colorectal carcinoma, indomethacin, celecoxib, PET imaging

## Abstract

Cyclooxygenase-2 (COX-2) is an important biomarker in several tumors. Available imaging probes display relatively low tumor to background ratios (smaller than 2:1). We evaluated newly developed indomethacin (Ind) derivatives for *in vivo* molecular imaging of COX-2 expressing carcinoma. Radioiodinated Ind derivatives Ind-NH-(CH2)4-NH-3-[I-125]I-Bz ([I-125]5), Ind-NH-(CH2)4-NH-5-[I-124/125]I-Nic ([I-124/125]6) and Ind-NH-(CH2)4-NH-5-[I-125]I-Iphth ([I-125]7) were prepared from the respective SnBu3-precursors (45–80% radiochemical yield; > 95% radiochemical purity). The cellular uptake of [I-125]5 and [I-125]6 correlated with COX-2 expression determined by SDS page/Western blot analysis. [I-125]5 was predominantly localized in the cell membrane while [I-125]6 was internalized and displayed a diffuse and favorable cytoplasmic distribution. In contrast, [I-125]7 showed only low uptake in COX-2 positive cells. Co-incubation with the COX-2 inhibitor Celecoxib led to an almost complete suppression of cellular uptake of [I-125]5 and [I-125]6. *In vivo* molecular imaging using positron emission tomography (PET) in SCID mice xenografted with COX-2^+^ (HT29) and COX-2^−^ (HCT116) human colorectal carcinoma cells was performed for [I-124]6. HT29 xenografts displayed a significantly higher uptake than HCT-116 xenografts (5.6 ± 1.5 vs. 0.5 ± 0.1 kBq/g, *P* < 0.05) with an extraordinary high tumor to muscle ratio (50.3 ± 1.5). Immunohistological staining correlated with the imaging data. In conclusion, the novel radioiodinated indomethacin derivative ([I-124/125]6) could become a valuable tool for development of molecular imaging probes for visualization of COX-2 expressing tumors.

## INTRODUCTION

Cyclooxygenase-2 (COX-2) represents an attractive target for molecular imaging due to its unique graded expression patterns in normal, inflamed and malignant tissues [1, 2]. In contrast to the COX-1 isoform, which is constitutively expressed in most physiological tissues, COX-2 transcription is induced by a wide spectrum of growth factors and cytokines in specific pathophysiological conditions. Both isoforms are localized in the endoplasmatic reticulum (ER) and in the nuclear envelope and convert arachidonic acid to prostaglandins and thromboxane, which mediate different responses within the immediate environment [3]. The overexpression of COX-2 was shown to be associated with carcinogenesis in different tumor entities (including colon, breast, lung, ovarian and prostate tumor) [4]. Thus, COX-2-targeted molecular imaging may be useful for the early detection of cancer. Additionally, COX-2 expression levels have been shown to be a prognostic marker for transformation from ductal carcinoma *in situ* to invasive growth and generation of metastases in breast cancer [5]. *In vivo* molecular imaging of COX-2 is therefore a promising aspect in individualized treatment approaches. The correlation between cancer progression and increased COX-2 expression furthermore supports the concept of molecular imaging of COX-2 expression for detection and staging of cancer.

**Scheme 1:**
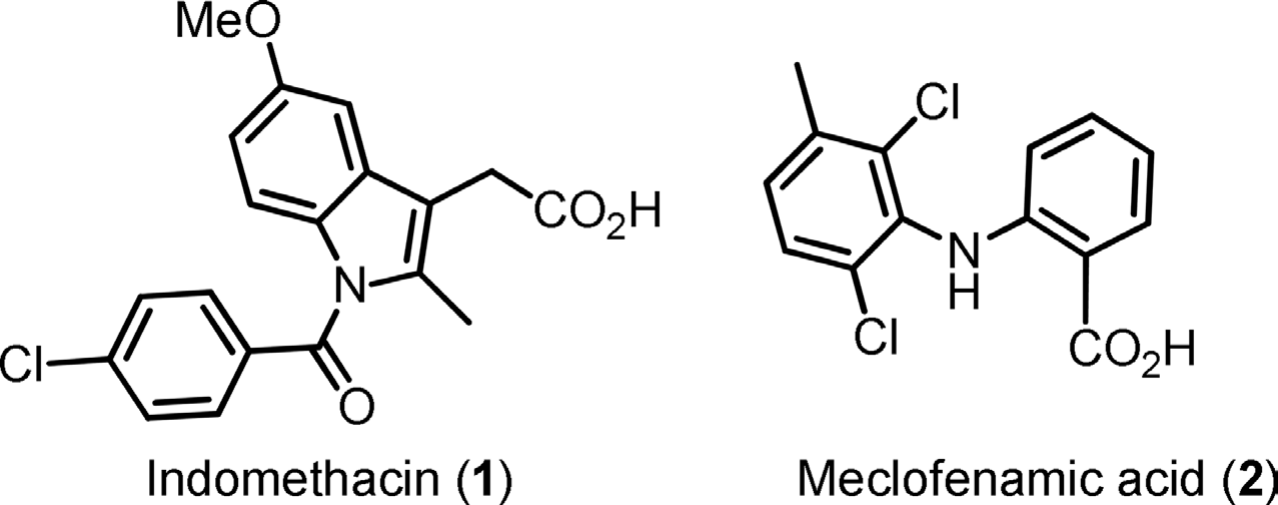
Structures of indomethacin (1) and meclofenamic acid (2)

**Scheme 2:**
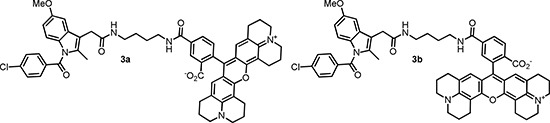
First fluorescent tracers suitable for visualization of COX-2 *in vivo*

Numerous COX inhibitors with different specificity and target affinity have been developed [6]. Traditional COX inhibitors such as Indomethacin 1 (Scheme 1) are non-selective and inhibit both isoforms of COX. A design of inhibitors selective for COX-2 seems to be rather difficult due to the high similarity of both enzyme isoforms [7]. Despite the high level of sequence homology between COX isoforms, substitutions at position Ile523, Ile434 and His513 in COX-1 by Val523, Val434 and Arg513 in COX-2 lead to structural variations within the catalytic domains. As a consequence of these alterations the COX-2 active site is about 27% larger than that of COX-1 [8, 9]. Importantly, the site residues at the active site channel are crucial for binding carboxylic acid-containing inhibitors by ion pairing and hydrogen bonding. Consequently, the transformation of the carboxylic group into ester or amide moieties converts moderately selective carboxylate-containing COX-1 inhibitors like indomethacin and meclofenamic acid (2) into COX-2 selective inhibitors [10]. Based on these findings Uddin et al. carried out extensive structure-activity relationship (SAR) studies of indomethacine derivatives conjugated with different fluorophores [11] and identified carboxy-x-rhodamines (ROX)-substituted indomethacine conjugates 3a and 3b containing 1,4-diaminobutane spacer between the pharmacophore and the fluorophore fragments as the first molecular probes suitable for *in vivo* detection of tissues with high level of COX-2 (Scheme 2) [12]. Accordingly, the 5-ROX-substituted conjugate 3a showed an up to 5-fold higher uptake in an inflamed rat paw compared to that in the contralateral non-inflamed paw. Furthermore, a significant accumulation of 3a in the COX-2 expressing 1483 HNSCC tumors in a mouse xenograft model was inhibited to > 90% by the pretreatment with indomethacin. At the same time the tracer uptake in HCT116 tumors which do not express COX-2 was minimal and independent of an indomethacin pretreatment.

The overall aim of this study was generation of novel tracers for visualization of COX-2 positive malignancies. Here we present the synthesis, chemical, biological *in vitro* and *in vivo* evaluation of novel radioiodinated indomethacin conjugates as probes for molecular imaging of COX-2 expressing tumors entities.

## RESULTS AND DISCUSSION

### Preparation of precursors and radiolabeling

**Scheme 3:**
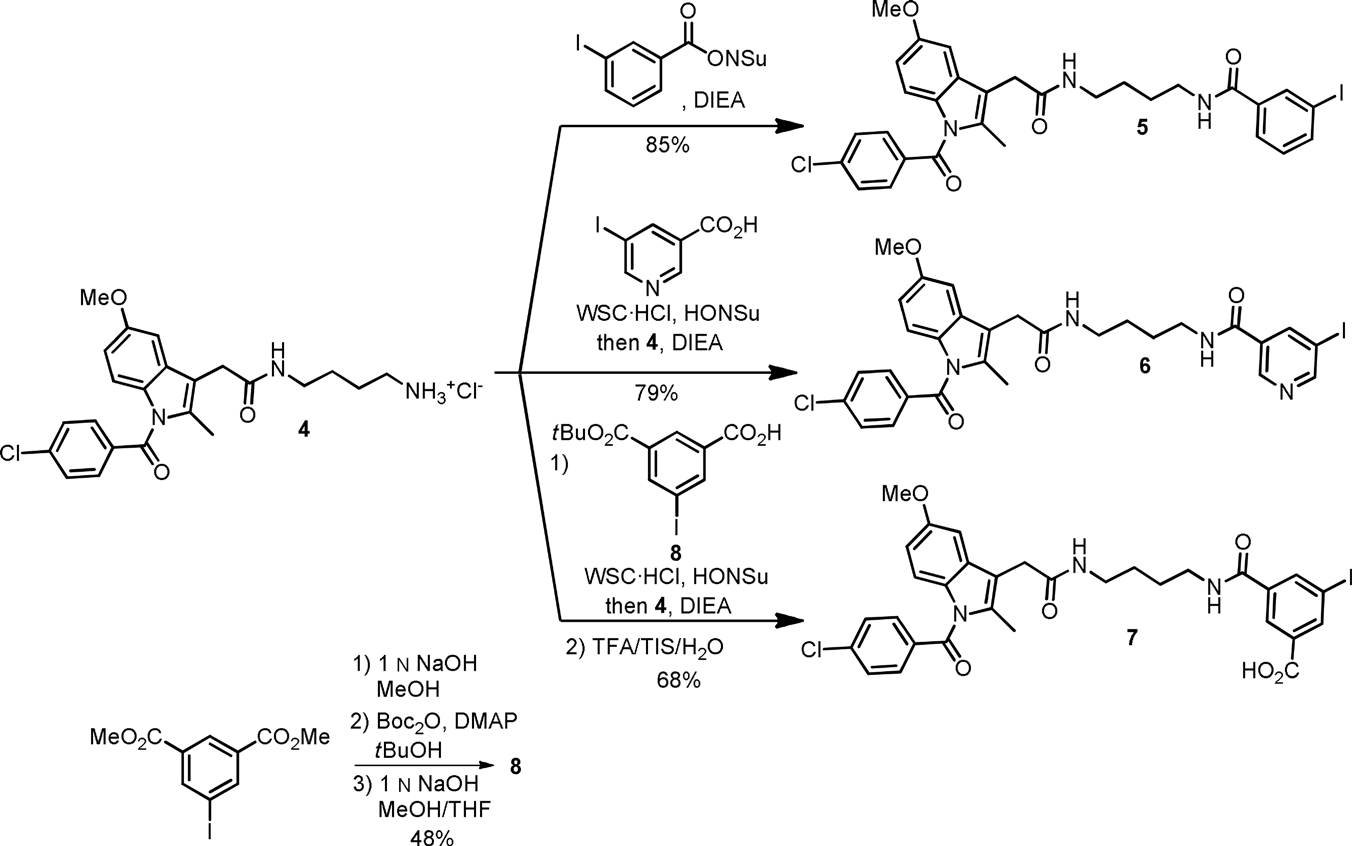
Preparation of indomethacine conjugates 5-7

**Scheme 4:**
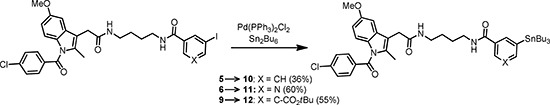
Preparation of radioiodination precursors 10-12

According to results of the SAR-study of fluorescent Indomethacin conjugates carried out by Uddin et al. [11] even large substituents like ROX-5 could be good tolerated by COX-2 provided that a sufficient length of the spacer between the pharmacophoric group and the reporter unit is ascertained. Consequently, three candidates of different lipophilicity and polarity were prepared. Distribution coefficients (Log D’s) determined according to the protocol of Donovan et al. [13] resulted in 4.76 ± 0.07, 4.41 ± 0.07 and 3.42 ± 0.08 for indomethacin amides 5, 6 and 7 (Scheme 3), respectively. Given log D values are valid for pH 6.8. The novel indomethacin substituted diamides 5–7 were prepared in 68–85% yield via acylation of Indomethacin-4-aminobutyl-1-amide (4) [14, 15] with the corresponding ONSu-esters (for the preparation of conjugate 6 the appropriate active ester was generated *in situ*) (Scheme 3). To prepare carboxy-substituted indomethacin derivative 7 an additional deprotection step of the intermediate *tert-*butyl ester 9 synthesized from 4 and mono-*tert-*butyl isophthalate (8) was necessary. The latter was prepared from dimethyl isophthalate in 48% yield over 3 steps as follows: hydrolysis into monomethyl isophthalate, preparation the corresponding *tert-*butyl methyl ester and, finally, cleavage of the residual methyl ester function (Scheme 3). The respective Bu3Sn-precursors for radioiodination 10-13 were obtained in 36–60% yield via [Pd]-catalyzed deiodostannylation (Scheme 4). Production of the COX-2 selective ligands 5 and 7 labelled with n.c.a. radiodine and of ligand 6 labelled with c.a. radioiodine as well as their purification and formulation in a small volume of a biocompatible medium were optimized. Thus, I-125- labeled compounds 5–7 and I-124- labeled compound 6 were produced in amounts sufficient for cell uptake and small animal μPET-experiments, respectively.

### Cellular binding studies

**Figure 1:**
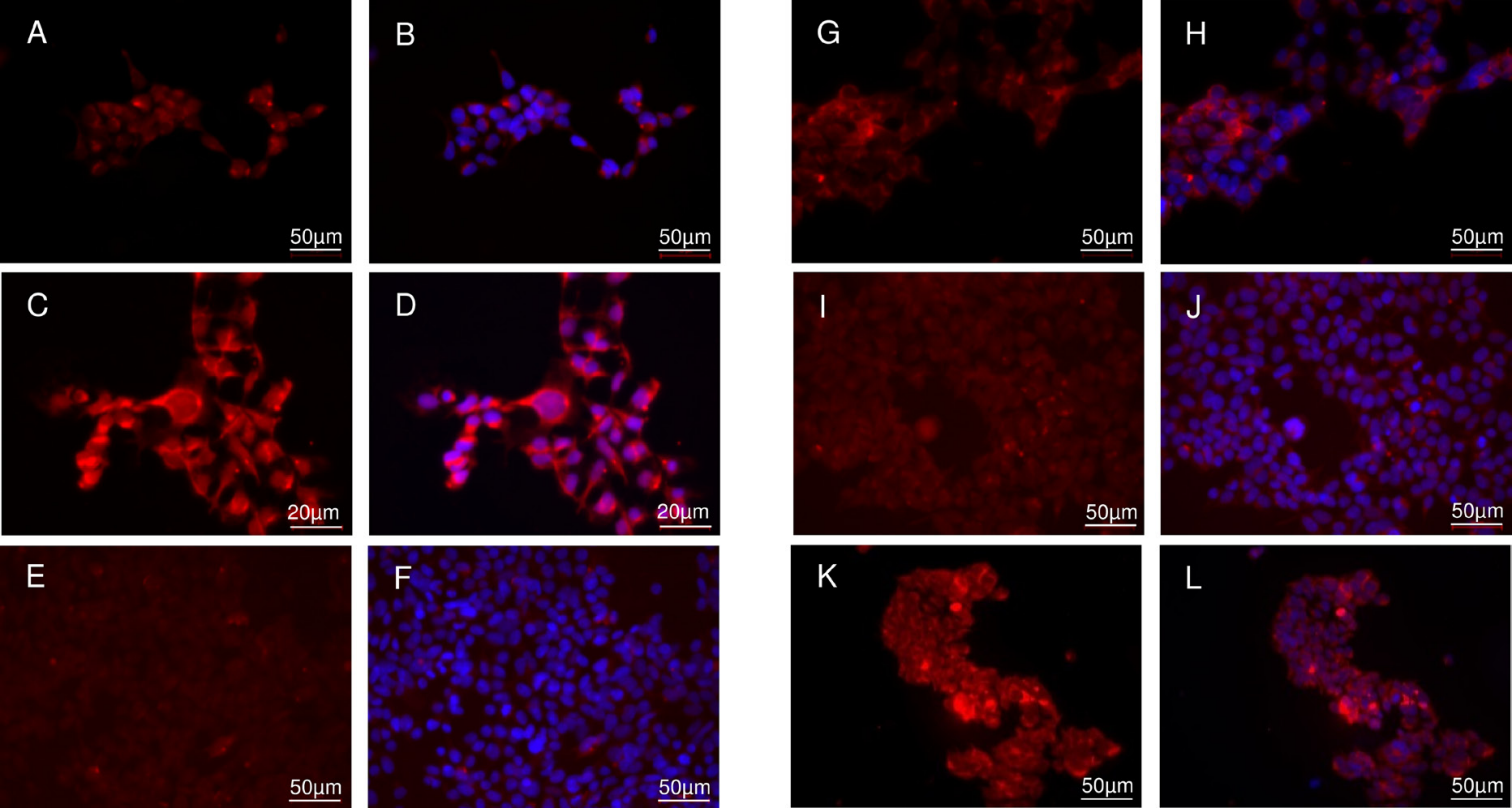
Cellular binding study in HEK *hCOX2del* cells Native staining in unstimulated (**A**, **B**) and Tet-stimulated (**C**–**L**) cells with ROX-5-Indomethacin. (A–D) ROX-5-Indomethacin staining without competitor. (E, F) ROX-5-Indomethacin staining with Celecoxib. (G, H) ROX-5-Indomethacin staining with compound 5. (I, J) ROX-5-Indomethacin staining with compound 6. (K, L) ROX-5-Indomethacin staining with compound 7. Nucleus counterstaining with DAPI. B, D, F, H, J and L are DAPI-overlaid images of A, C, E, G, I and K, respectively.

The candidates were first investigated for their ability to competitively displace the COX-2 bound fluorescent ROX-5-Indomethacin in HEK *hCOX-2del* cells (Figure 1). The staining of COX-2 by ROX-5-Indomethacin corresponded to Tet stimulation (Figure 1A–1D) and, consequently, confirmed COX-2 specificity of the probe. The incubation with ROX-5-Indomethacin led to perinuclear and ER membranous labeling, which correlated with the intracellular localization of COX-2 [16]. The co-incubation of the cells with the COX-2 selective inhibitor Celecoxib prevented the labeling by ROX-5-Indomethacin (Figure 1E, 1F). As indicated by remained membrane staining, compounds 5 and 7 were less potent than Celecoxib regarding displacement of ROX-5-Indomethacin (Figure 1G, 1H, 1K, 1L). This is probably due to the higher lipophilicity of compound 5 which, on the one hand, enables its passive transport through the cell membrane, but, on the other hand, contributes to unspecific retention in the lipid bilayer. The lack of displacement by compound 7 is probably attributed to its anionic character under physiological conditions, which hinders the passive transport through the negatively charged cell membrane. The compound 6 exhibited a high potency to displace ROX-5-Indomethacin binding to COX-2 (Figure 1I, 1J).

The tethered amides of certain carboxylic acid inhibitors such as indomethacin were suggested to selectively bind to COX-2 by breeching the protein domain which controls the access to the active site of COX-2 [10, 17]. In this study, the compound 6 showed a high COX-2 inhibitory activity comparable with that of Celecoxib. While lipophilic groups are supposed to be inherent for hydrophobic interactions with the COX binding pocket, in the case of compound 5 the high overall molecule lipophilicity contributes to its partial retention in the plasma membrane.

### Cellular uptake of radio iodinated Indomethacin derivatives

**Figure 2:**
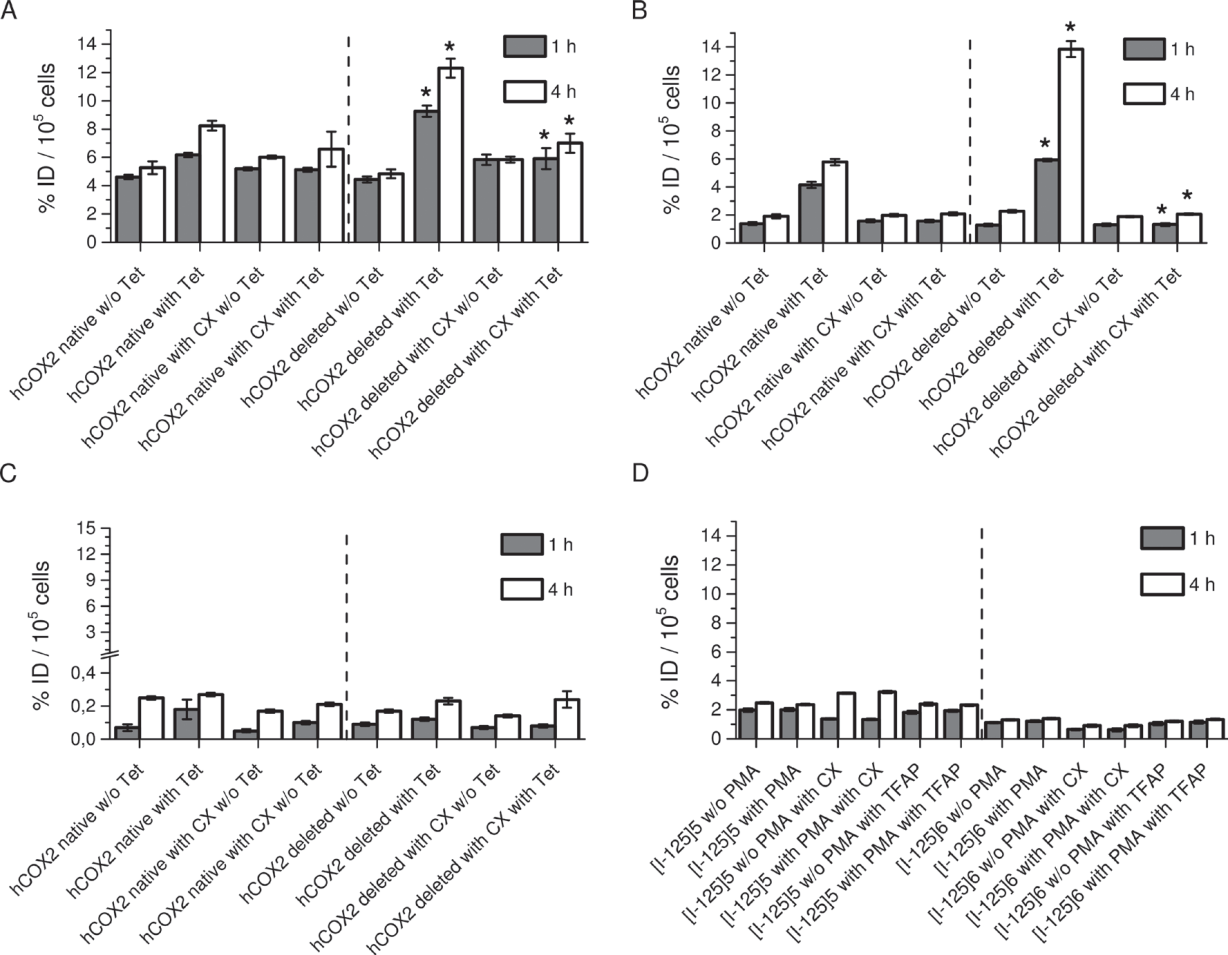
Cellular uptake experiments with radiolabeled compounds (**A**) Cellular uptake of [I-125]5 in unstimulated and Tet-stimulated HEK *hCOX-2nat* and HEK *hCOX-2del* cells w/o and with Celecoxib (CX). (**B**) Cellular uptake of [I-125]6 in unstimulated and Tet-stimulated HEK *hCOX-2nat* and HEK *hCOX-2del* cells w/o and with Celecoxib (CX). (**C**) Cellular uptake of [I-125]7 in unstimulated and Tet-stimulated HEK *hCOX-2nat* and HEK *hCOX-2del* cells w/o and with Celecoxib (CX). (**D**) Cellular uptake of [I-125]5 and [I-125]6 in unstimulated and PMA-stimulated HUVEC cells w/o and with TFAP. Data are mean (% of incubated dose (ID)/well) ± SD from three independent experiments. In A, B, *P < 0.05 by one-way ANOVA with Sidak´s multiple comparisons test.

**Figure 3:**
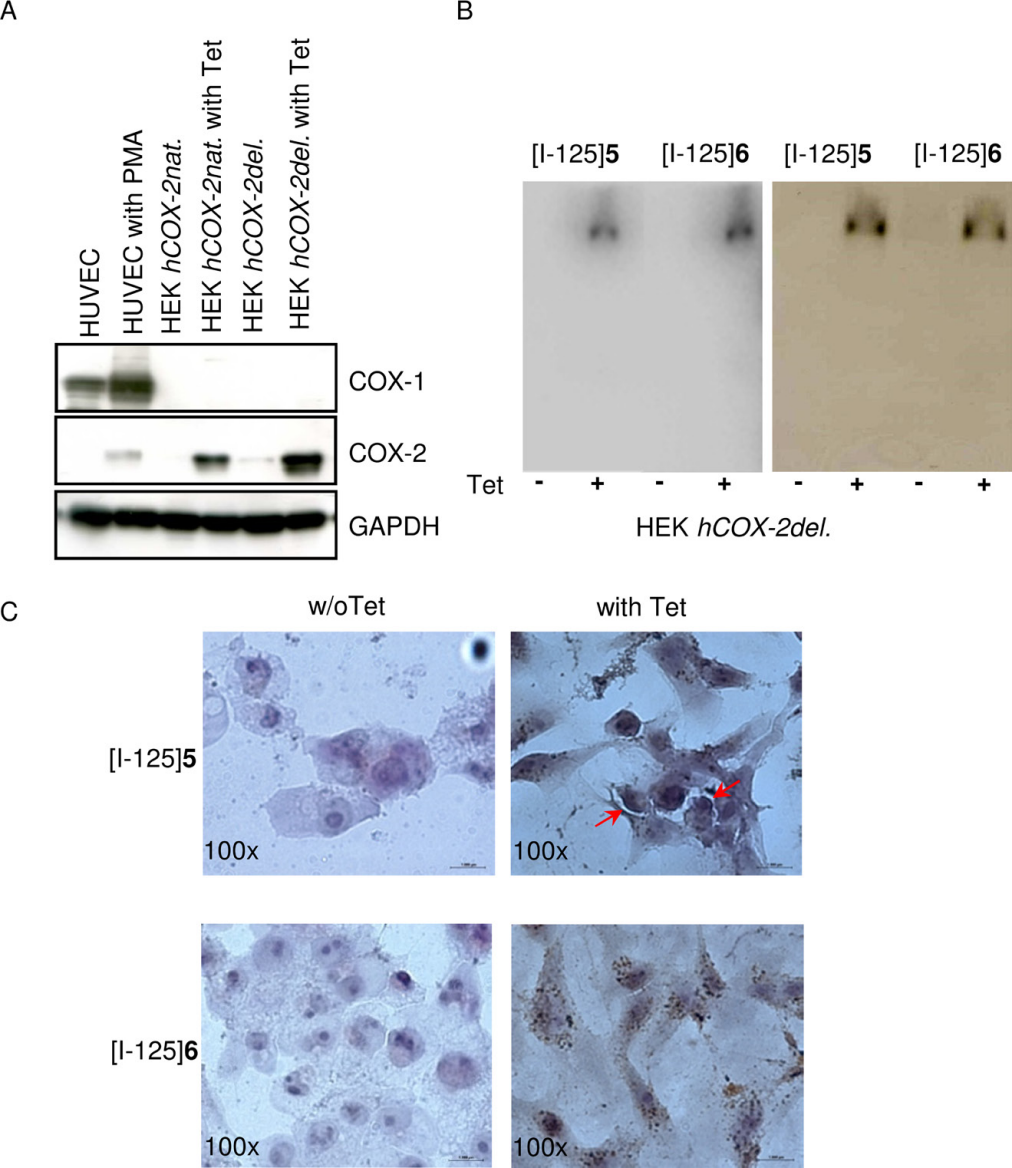
Binding specificity and intracellular distribution of iodinated indomethacin derivatives (**A**) SDS and western blot analysis of COX-1 and COX-2 expression in HUVEC, HEK hCOX-2*nat* and HEK hCOX-2*del* cells in dependency on PMA- and Tet-stimulation, respectively. GAPDH served as a loading control. (**B**) SDS gel electrophoresis of cell lysates obtained from HEK *hCOX-2del* cells incubated with [I-125]5 and [I-125]6 and visualized by phosphorimager (*left*); subsequent western blot analysis with COX-2 specific antibody. (**C**) Intracellular localization of [I-125]5 and [I-125]6 in HEK hCOX-2*del* cells detected by microautoradiography followed by a standard H&E staining. Arrows indicate the tracer localization.

To determine the passive membrane transport and COX-2 selective retention of the radiolabeled compounds in living cells, the uptake and blocking experiments were carried out in the intact cellular systems using HEK cells with inducible COX-2 expression and COX-1 positive HUVEC cells. The detected uptakes of the radiolabeled compounds [I-125]5 and [I-125]6 in the HEK cells raised in a time dependent manner (Figure 2A, 2B). For [I-125]5, the stimulation with tetracycline significantly increased the cellular accumulation in both HEK *hCOX-2nat* and HEK *hCOX-2del* cells (5.23 ± 0.46 vs. 8.24 ± 0.35% ID/well for HEK *hCOX-2nat* and 4.84 ± 0.31 vs.12.31 ± 0.66% ID/well for HEK *hCOX-2del* cells after 4 h incubation, respectively, *P* < 0.05). The higher uptake in HEK *hCOX-2del* cells corresponded to the stronger expression of C-terminally deleted COX-2 protein (Figure 3A). Similarly, in competition studies with ROX-5-Indomethacin the [I-125]5 compound retained unspecifically in the cell membrane. The co-incubation with the COX-2 specific inhibitor Celecoxib only partially blocked the [I-125]5 cellular retention (8.24 ± 0.35 vs. 6.58 ± 1.24% ID/well for HEK *hCOX-2nat* and 12.31 ± 0.66 vs. 7.00 ± 0.67% ID/well for HEK *hCOX-2del* cells, respectively, P<0.05). Compound [I-125]6 in contrast exhibited a very high and COX-2 specific cellular uptake and retention (1.91 ± 0.15% ID/well without Tet vs. 5.78 ± 0.23% ID/well with Tet for HEK *hCOX-2nat* cells and 2.27 ± 0.10% ID/well without Tet vs.13.85 ± 0.57% ID/well with Tet for HEK hCOX-2del cells, respectively, *P* < 0.05). The cellular uptake and retention of [I-125]6 was efficiently blocked by co-incubation with Celecoxib (5.78 ± 0.23% ID/well vs. 2.09 ± 0.10% ID/well for HEK hCOX-2nat and 13.85 ± 0.57% ID/well vs. 2.06 ± 0.06% ID/well for HEK *hCOX-2del* cells, respectively, *P* < 0.05). Corresponding to the staining study with ROX-5-Indomethacin [I-125]7 showed only marginal uptake in COX-2 expressing cells (Figure 2C). Importantly, none of the compounds showed a COX-1/COX-2 cross-selectivity (Figure 2D). Despite that PMA increased expression of COX-1 in HUVEC cells (Figure 3A), the cellular uptake of [I-125]5 and [I-125]6 in these cells remained unaffected and at low level (2.48 ± 0.05% ID/well vs. 2.36 ± 0.06% ID/well for [I-125]5 and 1.30 ± 0.05% ID/well vs.1.41 ± 0.07% ID/well for [I-125]6 without and with PMA stimulation, respectively). Blocking with Celecoxib and the COX-1 selective inhibitor TFAP [18] did not reduce the cellular uptake of [I-125]5 and [I-125]7. These findings support an unspecific trapping of both compounds in the cell membrane.

### Binding specificity and intracellular distribution of radioiodinated indomethacin derivatives [I-125]5 and [I-125]6

To determine the COX-2 specific cellular retention of [I-125]5 and [I-125]6, the protein-bound radioactivity in lysates obtained from HEK *hCOX-2del* cells was studied using a native SDS-gel electrophoresis followed by phosphor imager analysis (Figure 3B, left panel). For both compounds a significant protein-bound radioactivity fraction with distinct band at about 150 kDa was detected. This corresponded to the molecular weight of the reported dimerized form of COX-2 [19] und was further confirmed by a subsequent Western blot analysis with a COX-2 specific antibody (Figure 3B, right panel). By using microautoradiographic imaging the subcellular distributions of both probes could be determined (Figure 3C). [I-125]5 and [I-125]6 exhibited a perinuclear and diffuse cytoplasmic staining characteristic for COX-2 [16]. Noteworthy, [I-125]5 exhibited additional accumulation within the cell plasma membrane (Figure 3C indicated by narrows). This finding goes along with its unspecific retention in COX-2 negative cells as well as with the incomplete blocking of [I-125]5 uptake with Celecoxib. In conclusion, the reason for the different behavior of [I-125]5 and [I-125]6 most probably is due to their different lipophilicity, which in case of [I-125]5 is too high and not optimal for the effective permeation of the cell membrane.

### Molecular imaging of COX-2 expressing tumors

**Figure 4:**
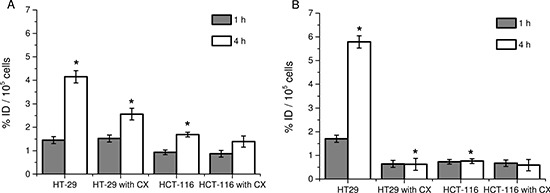
Cellular uptake with [I-125]5 and [I-125]6 compounds in colon carcinoma cells (**A**) Cellular uptake of [I-125]5 in HT29 and HCT-116 cells w/o and with Celecoxib (CX). (**B**) Cellular uptake of [I-125]6 in HT29 and HCT-116 cells w/o and with Celecoxib (CX). Data are mean (% of incubated dose (ID)/well) ± SD from three independent experiments. *P < 0.05 by one-way ANOVA with Sidak´s multiple comparisons test.

The potential of [I-125]5 and [I-125]6 as tracer for the detection of COX-2 expressing tumors was investigated in COX-2 positive HT29 cells [20] and COX-2 negative HCT-116 cells [21] derived from human colorectal carcinomas. In a preliminary *in vitro* study, both compounds have been shown to accumulate specifically in COX-2 expressing tumor cells (4.15 ± 0.26% ID/well vs. 1.69 ± 0.12% ID/well in HT29 cells vs. HCT-116 for [I-125]5 and 5.79 ± 0.26% ID/well vs. 0.76 ± 0.10% ID/well in HT29 cells vs. HCT-116 for [I-125]6, *P* < 0.05) (Figure 4A, 4B). Summarizing the entire *in vitro* studies, compound 6 displayed a higher degree of selectivity of cellular uptake and retention in COX-2 expressing tumor cells compared to compound 5.

**Figure 5:**
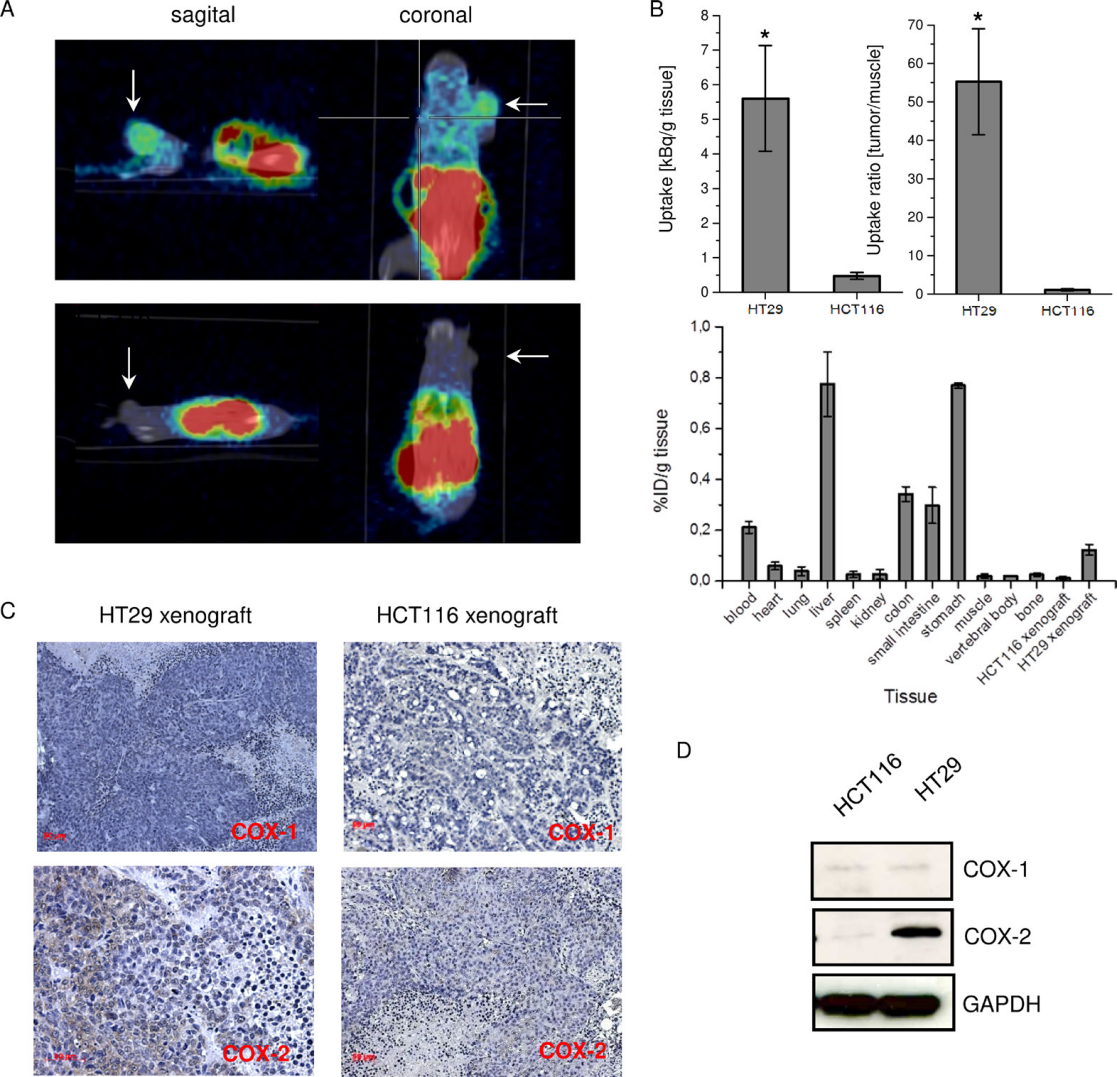
*In vivo* study with [I-124]6 compound in colon carcinoma xenografted SCID mice (**A**) μPET/CT molecular imaging of COX-2 with [I-124]6 in HT29 (upper panel) and HCT-116 (lower panel) xenografted SCID mice at 4 h p.i. Arrows indicate tumor. (**B**) Uptake of [I-124]6 in HT29 and HCT-116 tumors (in kBq/g tissue) (upper panel, left); tumor to muscle uptake ratio of [I-124]6 in HT29 and HCT-116 tumor xenografted SCID mice (upper panel, reight) at 4 h p.i.; gamma counter analysis of [I-124]6 biodistribution in HT29-xenografted SCID mice at 4 h p.i. (in %ID/g tissue) (*n* = 5). (**C**) Immunhistological analysis of COX-1 and COX-2 in HT29 and HCT-116 xenografts. (**D**) Corresponding SDS and Western Blot analysis of COX-1 and COX-2 expression in HT29 and HCT-116 xenografts. Data are mean ± SD from five independent experiments. In B, *P < 0.05 by two-tailed Student´s *t-test*.

Based on the *in vitro* indications of superior COX-2 binding characteristic, [I-124]6 was evaluated in HT29 and HCT-116 xenografted SCID mice regarding its potential of for molecular imaging of COX-2 expressing tumors *in vivo*. At 4 h post injection (~5 MBq [I-124]6), the animals were placed in the μPET for evaluating the tracer biodistribution. The radioactivity was localized mainly in the liver and in the gastrointestinal tract (Figure 5A). This can be assigned to the lipophilicity of the tracer and its associated hepatobiliary extraction route. Importantly, a significant uptake of [I-124]6 was detected in the HT29 xenograft (Figure 5A, 5B, *P* < 0.05). In contrast, in the HCT-116 xenograft virtually no [I-124]6 accumulation was observed. After μPET and CT imaging the tissues were removed, weighted and analyzed regarding the accumulated radioactivity in a gamma counter. The overall biodistribution analysis confirmed the hepatobiliary excretion and prolonged blood retention of [I-124]6 compound (Figure 5B, lower panel). The normalized tissue uptake of [I-124]6 (kBq/g tissue) was approximately 5-fold higher in the COX-2 expressing HT29 tumor than in COX-2 negative HCT-116 tumor (Figure 5B, upper panel, left). In an earlier study with a different compound in a comparable tumor model the ratio of uptake between COX-2^+^ versus COX-2^−^ tumors was as low as 3 [22]. Analogue to previous studies presenting COX-2 addressing probes the tracer retention in the tumor tissue was calculated relatively to the uptake in muscle as a reference tissue [22, 23]. For [I-124]6 the tumor/muscle ratio was more than 50-fold higher in HT29 compared to HCT-116 xenografted mice (Figure 5B, upper panel, right).

The immunohistological staining analysis with COX-1 and COX-2 specific antibodies verified that the difference in uptake of [I-124]6 in HT29 and HCT-116 tumors is attributed to the distinct expression pattern of COX-2 (Figure 5C, 5D). Since the *in vitro* result demonstrated the specific uptake of [I-124]6 by a competition experiment and since the immunochemistry confirmed the co-localization of uptake and COX-2 expression no *in vivo* competition experiments were carried out. However, for more detailed *in vivo* characterization, the [I-124]6 should be evaluated in conditional knockout mouse for tissue-specific disruption of COX-2 gene [24, 25].

The correlation of elevated COX-2 expression with chronic inflammation and pre-malignancy and cancer progression [5, 26] suggests COX-2 as a useful target for imaging early tumor lesions. Moreover, because inflammation often reflects the predisposition to malignancy the COX-2 addressing tracer may enable detection of high-risk premalignant lesions and guide preventive interventions. Therefore, in this study we developed PET imaging probes which can serve as tracers for early detection, risk assessment and prognosis of COX-2 expressing malignancies. For all three tracers [*I]5–7, production routes have been optimized in terms of radiochemical yields and formulation in a small volume of a biocompatible medium. However, in case of [*I]6, addition of a small amount of carrier iodine which did not affect its binding affinity was necessary to achieve reasonable radiochemical yields. The uptake and competition studies using an intact cellular system in combination with protein binding analysis as well as microautoradiography of incubated cells demonstrated a high COX-2 affinity of two out of the three candidates (5 and 6). The more polar compound 6 showed the most favorable pharmacological profile *in vitro* and *in vivo* with high selective accumulation in COX-2 expressing tumor cells and negligible uptake in COX-2 negative cells. In comparison to other already published probes, the tumor to muscle ratio of [*I]6 is very high [22, 23]. Conclusively, considering the overall low accumulation in the tumor tissue of about 0.1% of ID/g tissue and the superior tumor to muscle ratio compound 6 represents a promising leader structure for development SPECT and PET imaging probes (depending on the radioiodine isotope) for the detection of COX-2 expressing tumors in clinical settings.

## MATERIALS AND METHODS

### Cell line and cell culture reagents

Human embryonic kidney (HEK)-293-derived cell lines expressing human COX-2 constructs (HEK *hCOX-2nat* and HEK *hCOX-2del*) were used for the *in vitro* experiments (kindly provided by Prof. W. Smith (Michigan, USA)). The mutated form of COX-2 was constructed by deletion of 18 AAs inside the C-terminus, which were shown to be responsible for rapid protein degradation [16]. This mutant maintains the activity and binding specificity of native COX-2 but shows prolonged biological half-life (t_1/2_ ~2 h vs. t_1/2_ > 24 h, for native and mutated COX-2, respectively). Transfected HEK293 cells were cultured in DMEM (Life Technologies, Gibco, Carlsbad, USA) containing 10% FCS (Biochrom, Berlin, Germany), 100 units/mL of penicillin and 100 μg/mL of streptomycin (Life Technologies). For induction of COX-2 expression cells were serum-starved for 24 h and subsequently incubated in serum-free medium containing 10 μg/mL tetracycline (Sigma Aldrich, St. Louis, USA). Human colon carcinoma HT29 and HCT-116 cell lines were obtained from LGS Standards (Teddington, UK) and grown in McCoy's 5A medium (Biochrom) containing 10% FCS, 100 units/mL of penicillin and 100 μg/mL of streptomycin. The umbilical vein/vascular endothelial cells HUVEC were purchased from LGS Standards and cultured in F-12K Medium (Biochrom) supplemented with 0.1 mg/mL heparin (Biochrom), 0.05 mg/mL endothelial cell growth supplement (Sigma-Aldrich), 10% FBS, 100 units/mL of penicillin and 100 μg/mL of streptomycin. ROX-5-labeled indomethacin was synthesized as previously described [11, 12]. The COX-1 specific inhibitor N-(5-Amino-2-pyridinyl)-4-trifluoromethylbenzamide (TFAP) and the COX-2 specific inhibitor Celecoxib (CX) were purchased from Merck and Sigma Aldrich, respectively. The cell lines were authenticated by the STR profiling. All the cell cultures were tested for Mycoplasma contamination by PCR before use.

### Chemical preparations

Syntheses and characterizations of all compounds are described in the Supplementary Data.

### Determination of log D values

The log D values were assessed at least in triplicate using a HPLC based method described by Donovan et al. [13] using the internal standards toluene (Log D = 2.72) and triphenylene (Log D = 5.49). The used analytical column was: Asahipak ODP-50G, 4.6 × 10 mm (Showa Denko Europe GmbH (Shodex)) applying a gradient elution: 0.5 min à 8.0 min from 10 %A à 100 % A (keeping for 4 min); with eluent A = MeOH, B = 25 mM sodium hydrogen phosphate aq, pH 6.8. The flow was 2 mL/min and UV was set at 260 nm. Log D values equalized with log *P* values were calculated from measured retention times using formula (4) [13]. A typical chromatogram is supplied within the supplementary information.

### Radiolabeling

The procedure for the radiosynthesis is described in the Supplementary Data. The following three compounds were synthesized: Ind-NH-(CH2)4-NH-3-[I-125]I-Bz ([I-125]5), Ind-NH-(CH2)4-NH-5-[I-124/125]I-Nic ([I-124/125]6) and Ind-NH-(CH2)4-NH-5-[I-125]I-Iphth ([I-125]7).

*In vitro* intact cell competition assay

Non-stimulated and Tet-stimulated HEK293 *hCOX-2del* cells were incubated in serum-free medium with ROX-5-labeled Indomethacin (200 nmol/L) with or w/o the competitor Celecoxib (CX), compounds 5, 6 and 7 (each 5 μmol/L) for 45 min at 37°C. The cells were washed thrice and incubated in fresh serum-free medium for 45 min (wash-out step). Harvested cells were fixed in 2% paraformaldehyde. For microscopy analysis, nuclear counterstaining was accomplished with Hoechst33342 (1 mg/mL), before being examined by fluorescence microscopy (ZEISS, Axio Scope A1).

### Cellular uptake with radio iodine labeled Indomethacin derivatives

To investigate the COX-2 specificity, the cells (1*10^5^/well) were seeded into 12-well plates. I-125-labeled Ind derivatives were added to the cells (0.2 MBq/well in the presence of 0.1 nmol/L of the corresponding carrier) with or without CX (5 μmol/L) and incubated for 1 h and 4 h at 37°C and 5% CO_2_. To investigate the COX-1 specificity, non-stimulated and phorbol-12-myristate-13-acetate stimulated (PMA, 10 nmol/L for 24 h, Sigma Aldrich) HUVEC cells (1*10^5^/well) were seeded into 12-well plates. I-125-labeled Ind derivatives were added to the cells (0.2 MBq/well in the presence of 0.1 nmol/L of the corresponding carrier) with or without COX-1 inhibitor TFAP (5 μmol/L) and incubated for 1 h and 4 h at 37°C and 5% CO_2_. After incubation, the medium was removed; the cells were washed with fresh medium and incubated for a further hour at 37°C and 5% CO_2_. The cellular uptakes were measured using a gamma counter (Wizard2, Perkin-Elmer).

### Microautoradiography

Non stimulated and Tet-stimulated HEK293 *hCOX-2del* cells were incubated in serum-free medium with [I-125]5 and [I-125]6 (0.2 MBq) for 4 h at 37°C. The cells were washed thrice and incubated in fresh serum-free medium for 1 h. After fixation in 4% paraformaldehyde the slices were shortly dipped into a melted photographic emulsion (Kodak Emulsion NTB-2) and allowed to dry for 15 min in a darkroom. After an exposition period of 2 weeks at −20°C, the slices were developed (Kodak D-19 Film Developer) and fixed (Superfix Plus; Tetenal) followed by standard H&E staining. The cellular distribution of silver grains indicating radioactive sites was examined using bright field microscopy (Zeiss, Axio Scope.A1).

### Analysis of COX-1 and COX-2 expression

For analysis of COX-1 and COX-2 expression, cells and tissues were lysed with Tris·HCl buffer [50 mM, pH 7.4, NaCl (300 mM), EDTA (2 mM)], NP-40 (1%), PMSF (1 mM) and inhibitor cocktail (Roche). The protein samples were boiled for 5 min in reducing Laemmli buffer supplemented with 5% 2-mercaptoethanol. Equal amounts of protein were subjected to electrophoresis (4-20% Tris·HCl gel, BioRad) and blotted onto PVDF membrane (BioRad). Immunostaining of COX-1 was performed with goat polyclonal anti-COX-1 antibody and immunostaining of COX-2 with rabbit polyclonal anti-COX-2 antibody (both 1:1000, abcam, Cambridge, UK). The secondary anti-goat and anti-rabbit horseradish peroxidase coupled antibodies (both 1:2000, Cell Signaling, Cambridge, UK) were visualized with enhanced chemoluminescence (ECL+, GE Healthcare, UK) as recommended by the supplier. Equal protein loading was controlled using GAPDH specific antibody (1:1000, Cell Signaling) and secondary anti-rabbit antibody linked to horseradish peroxidase.

### Analysis of the derivatives–COX-2 interaction

For analysis of the Ind derivatives–COX-2 interaction, the total cell lysates of non-stimulated and Tet-stimulated HEK293 *hCOX-2del* cells were prepared after incubation with radioiodine labeled compounds following the procedure described above for SDS-PAGE/immunoblot analysis. The samples were prepared in non-reducing Laemmli buffer. For protein separation, equal amounts of each sample were subjected to electrophoresis (4-20% Tris·HCl gel). Subsequently, the gel was exposed overnight to a phosphorimager screen and visualized using phosphorimager (Personal Molecular Imager FX, Biorad, California, USA).

### Tumor model

A suspension of 2*10^6^ HT29 and HCT-116 cells was injected subcutaneously in to the scruff of the neck between the shoulders of 6 weeks old SCID mice (C.B-Igh-1b/IcrTac-Prkdcscid, Taconic, Hudson, USA). After 2 weeks, the mice developed subcutaneous tumors of about 15–20 mm diameter. All animal procedures and experiments were performed in accordance with the guidelines of the German Regulations of Animal Welfare. The protocols were approved by the local Ethical Committee for Animal Experiments.

### Small animal studies

Five MBq of [I-124]6 in isotonic saline containing 5% EtOH (200 μL) were injected into the lateral tail vein of SCID mice bearing xenotransplanted HT29 (*n* = 5) and HCT-116 (*n* = 5) tumors. Four hours later, the mice were placed in the microPET (Inveon, Siemens, Knoxville, USA) and imaged for 30 min (5% isoflurane anesthesia with 1.5% initial). CT images were produced by a Philips Gemini TF16 PET/CT (Philips Medical Systems, PC Best, The Netherlands). The CT scans enable anatomical co-localization of PET acquired biodistribution. The PET images were reconstructed using the iterative OSEM3D/MAP (OSEM3D 2 iterations, MAP 18 iterations) algorithm. Additionally, following the CT measurement the tumors and organs were excised, weighed and assayed for radioactivity using the gamma counter. Mean tumor and organ uptake was determined from decay-corrected tissue radioactivity normalized to injected dose and tissue sample weight (unit: % injected dose/g tissue wet weight: %ID/g).

### Immunohistochemistry of tumor tissue sections

Consecutive formalin-fixed, paraffin-embedded tissue sections (2 μm thick) were dewaxed in xylene and rehydrated through graded concentrations of ethanol to distilled water. Sections were then immersed in 10 mmol/L citrate buffer (pH 6.0) and processed in thermostatic water bath for 30 minutes at 98°C for antigen retrieval. After the antigen retrieval treatment, the tissue sections were incubated for 60 minutes with anti-COX-1 (1:1000) and anti-COX2 (1:1000) antibodies. Subsequently, the sections were exposed for 60 minutes to peroxidase-linked antibodies (1:200). Colour development was performed by using diaminobenzidine substrate. Sections were counterstained with hemalaun.

### Statistical analysis

Cellular uptake experiments were performed in triplicate and by repeating independent blocks of experiments. Data are presented as mean ± standard deviation. All statistical calculation were performed using Graph Pad Prism version 6.00 (San Diego California USA) for Windows. Data were analysed by Student´s *t-test* and one-way ANOVA with Post-hoc comparisons were performed with Sidak´s multiple comparison test. Effects were considered to be statistically significant if *P* ≤ 0.05.

## SUPPLEMENTARY INFORMATION


