# Supplementary material for: Radioiodinated indomethacin amide for molecular imaging of cyclooxygenase-2 expressing tumors
Source: Oncotarget. 2017 Feb 17;8(11):18059–69. doi: 10.18632/oncotarget.15437 (PMC5392307; doi:10.18632/oncotarget.15437)
Supplement: Supplementary file 1 [file oncotarget-08-18059-s001.pdf]

Radioiodinated indomethacin amide for molecular imaging of cyclooxygenase-2 expressing tumors

Supplementary Materials

SUPPLEMENTARY INFORMATION

MATERIALS AND METHODS

^{1}H-NMR spectra: Bruker Avance II 300 (300 MHz) and Bruker Avance II+ 600 (600 MHz). ^{1}H chemical shifts are reported in ppm relative to residual peaks of deuterated solvents. Higher-order NMR spectra were approximately interpreted as first-order spectra, where possible. The observed signal multiplicities are characterized as follows: s = singlet, d = doublet, t = triplet, q = quartet, quin = quintet, m = multiplet, and br = broad. Coupling constants (*J*) were reported in Hertz (Hz). ^{13}C-NMR spectra [additional APT (Attached Proton Test)]: Bruker Avance II 300 (75.5 MHz) and Bruker Avance II+ 600 (125.9 MHz). ^{13}C chemical shifts are reported relative to residual peaks of deuterated solvents. Low resolution ESI-MS: Finnigan LCQ. High resolution ESI-MS: Bruker APEX IV 7T FTICR MS. TLC: Merck pre-coated sheets, 0.25 mm Sil G/UV_{254}. The chromatograms were viewed under UV light and/or by treatment with phosphomolybdic acid (10% in ethanol). Column chromatography: Merck silica gel, grade 60, 230–400 mesh. Solvent proportions are indicated in a volume/volume ratio. All reactions were carried out with magnetic stirring unless otherwise stated and, in the case of air- or moisture-sensitive substrates and/or reagents, were handled in flame-dried glassware under argon or nitrogen. Organic extracts were dried with anhydrous $MgSO_4$. In general, chemicals and solvents were purchased from Sigma-Aldrich and Merck (Germany), or otherwise as indicated. In general, all reagents and solvents were of the highest commercially available grade and were used without further purification. Chloramine T trihydrate (CAS no. 7080-50-4) was from Sigma-Aldrich (product no. 31224), acetonitrile in HPLC grade for labeling was from Merck (product no. 114291). PBS (w/o Ca, Mg) was from PAN-Biotech. Used HPLC colums were "Multospher C2" (Multospher 120 RP 2, 5 µm, 250 × 3 mm, SC-Chromatographie Service, Germany), and "Chromolith" (monolithic Chromolith FastGradient RP-18 endcapped 50 × 2 mm, Merck Germany, product no. 152007). The used "C18 cartridge" was "Sep-Pak C18 Plus Light" (Waters, 130 mg sorbent, 55-105 µm particle size, product no. WAT023501). No-carrier-added (n.c.a.) sodium [I-124]iodide ($A_s \leq 1203$ GBq/µmol, $A_c > 1$ MBq/µL, in 0.02 M NaOH) was obtained from Eckert & Ziegler (Berlin, Germany), and no-carrier-added sodium [I-125] iodide (Product NEZ033H010MC, $A_s \geq 79.9$ GBq/µmol, $A_c \geq 13$ MBq/µL, in 0.01 M NaOH) was purchased from PerkinElmer. Radiochemical yields (RCY) were calculated as the amount of radioactivity in the product expressed as the percent of related starting radioactivity used in the corresponding synthesis (step). The necessary decay correction was omitted in consideration of the long half-lives of the nuclides used [Ref.: http://www.srsweb.org/]. Further used abbreviations are: RCP for radiochemical purity, and EOS for end of 4 synthesis.

Indomethacyl pentaflurophenolate, compound 3a, *N*-(4-aminobutyl)-2-(1-(4-chlorobenzoyl)-5-methoxy-2-methyl-1*H*-indol-3-yl)acetamide hydrochloride (4), *N*-hydroxysuccinimidyl 3-iodobenzoate and 5-iodopyridine-3-carboxylic acid were prepared according to the literature [1–5].

Chemistry

Determination of log D values

For determining log D values using the HPLC method described within the Materials and Methods section of the corresponding article synthetic reagent mixtures were at least analyzed in triplicate. The basic cocktail was composed by adding 2 mg of triphenylene to 200 µL of toluene, followed by the addition of 20 mL methanol. For assessing the log D of a substance approximately 1 mg of it was added to 1 mL of the cocktail. Compound 7 was dissolved partly only. 5 µL of each compound solution were injected into the HPLC. A typical chromatogram with all three investigated COX-2 inhibitors plus indomethacin is shown in the following chromatogram (Supplementary Figure 1).

Indomethacin conjugate 5 [Ind-NH-$(CH_2)_4$-NH-3-I-Bz]

Diisopropylethylamine (0.193 mL, 0.14 g, 1.08 mmol) was added to a suspension of *N*-(4-aminobutyl)-2-(1-(4-chlorobenzoyl)-5-methoxy-2-methyl-1*H*-indol-3-yl) acetamide hydrochloride (4) (0.25 g, 0.54 mmol) in a solution of *N*-hydroxysuccinimidyl 3-iodobenzoate (0.186 g, 0.54 mmol) in DMF (5 mL) and the mixture was stirred for 3 h. Thereafter, the mixture was concentrated

under reduced pressure, the residue dissolved in EtOAc (80 mL) and washed with 1 M $NaHSO_4$ (3 × 20 mL), H_2O (20 mL), 10% $NaHCO_3$ (3 × 20 mL), H_2O (3 × 20 mL), brine (2 × 10 mL), dried, filtered and concentrated under reduced pressure. The residue purified by column chromatography ($CHCl_3$:MeOH = 25:1) and trituration with Et_2O to give 5 (0.29 g, 85%) as a colorless solid. R_f = 0.21, $CHCl_3$:MeOH = 25:1. ^{1}H NMR (300 MHz, $CDCl_3$) δ 8.22–8.01 (m, 1H), 7.88–7.67 (m, 2H), 7.69–7.58 (m, 2H), 7.52–7.40 (m, 2H), 7.22–7.06 (m, 1H), 6.94–6.88 (m, 1H), 6.87–6.81 (m, 1H), 6.71–6.65 (m, 2H), 6.04–5.89 (br, 1H), 3.78 (s, 3H), 3.65 (s, 2H), 3.43–3.34 (m, 2H), 3.31–3.16 (m, 2H), 2.38 (s, 3H), 1.66–1.50 (m, 4H).^{13}C NMR (75 MHz, $CDCl_3$) δ 170.4, 168.5, 166.1, 156.3, 140.4, 139.8, 136.64, 136.56, 136.2, 133.7, 131.3, 131.1, 130.5, 130.3, 129.4, 126.3, 115.26, 112.9, 112.3, 101.3, 94.3, 56.0, 39.8, 39.2, 32.4, 27.1, 26.6, 13.4. ESI HRMS: calcd for $C_{30}H_{29}N_3O_4ClINa^+$: 680.0783; found: 680.0777; calcd for $C_{30}H_{28}N_3O_4ClI^-$: 656.0808; found: 656.0811.

Indomethacin conjugate 6 (Ind-NH-(CH_2)$_4$-NH-5-I-Nic)

WSC·HCl (0.27 g, 1.41 mmol) was added to an ice-cold solution of 5-iodopyridine-3-carboxylic acid (0.29 g, 1.16 mmol) and HONSu (0.16 g, 1.39 mmol) in DMF (5 mL). Thereafter, CH_2Cl_2 was added to the reaction mixture until WSC·HCl completely dissolved. The cooling bath was removed and the mixture was stirred for an additional 1 h. After that, a freshly prepared solution of 4 (0.55 g, 1.04 mmol) and diisopropylethylamine (0.175 mL, 0.13 g, 1.11 mmol) in DMF (10 mL) was added to the solution of the active ester and the reaction mixture was stirred for a further 3 h. Thereafter, the mixture was concentrated under reduced pressure, the residue dissolved in CH_2Cl_2 (80 mL) and washed with H_2O (3 × 20 mL), brine (2 × 10 mL), dried, filtered and concentrated under reduced pressure. The residue was taken up in EtOAc (30 mL) and the resulting suspension was heated to reflux and cooled down to ambient temperature. The precipitate was filtered off to give 7 (0.54 g, 79%) as a colorless solid. R_f = 0.19, $CHCl_3$:MeOH = 20:1 (two times development). ^{1}H NMR [300 MHz, $(CD_3)_2SO$] δ 8.88–9.02 (m, 2H) 8.68 (t, J = 5.2 Hz, 1H) 8.52 (t, J = 2.1 Hz, 1H) 7.98–8.08 (m, 1H) 7.58–7.73 (m, 4H) 7.11 (d, J = 2.1 Hz, 1H) 6.93 (d, J = 9.0 Hz, 1H) 6.69 (dd, J = 9.0, 2.3 Hz, 1H) 3.74 (s, 3H) 3.49 (s, 2H) 3.25 (q, J = 5.7 Hz, 2H) 3.03–3.15 (m, 2H) 2.22 (s, 3H) 1.48 (m, 4H).^{13}C NMR [75 MHz, $(CD_3)_2SO$] δ 169.2, 167.8, 163.3, 157.1, 155.5, 147.0, 142.5, 137.5, 135.0, 134.2, 131.5, 131.1, 130.9, 130.2, 129.0, 114.5, 114.4, 111.2, 101.8, 93.9, 55.4, 31.2, 26.6 (×2), 26.3 (×2), 13.3. MS (ESI): positive mode m/z = 1339.1 ([2M + Na]$^+$), 681.0 ([M + Na]$^+$), 659.1 ([M + H]$^+$); MS (ESI): negative mode m/z = 657.1 ([M – H]$^-$); ESI HRMS: calcd for $C_{29}H_{29}N_4O_4ClINa^+$: 659.0917; found: 659.0911; calcd for $C_{29}H_{27}N_4O_4ClI^-$: 657.0771; found: 657.0760.

Mono-tert-butyl 5-iodoisophthalate (8)

1 M NaOH (3.4 mL) was added to a solution of dimethyl 5-iodoisophthalate (1 g, 3.12 mmol) in MeOH (10 mL) and the reaction mixture was stirred for 16 h. Afterwards, the mixture was concentrated under reduced

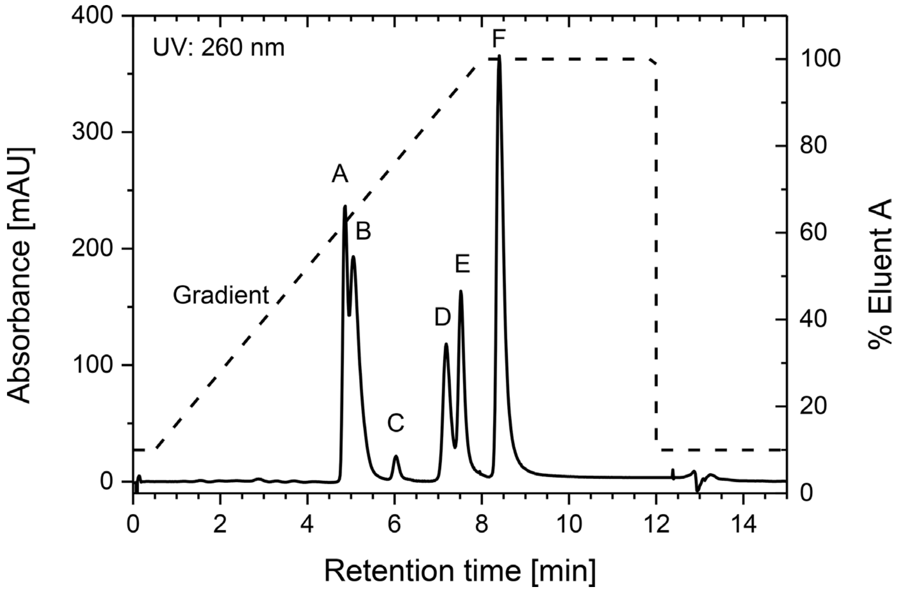

Supplementary Figure 1: HPLC chromatogram of a synthetic mixture of pure substances: A: indometacin, B: toluene (internal standard), C: compound 7, D: compound 6, E: compound 5, F: triphenylen (internal standard). Column: Asahipak ODP-50G, 4.6 × 10 mm (Showa Denko Europe GmbH (Shodex)), Eluent A = MeOH, B = 25 mM sodium hydrogen phosphate aq, pH 6.8. Flow: 2 mL/min, UV at 260 nm. Gradient as shown (dashed line). Loop: 500 µL, Injection volumes: 5 µL (cocktail plus compound).

pressure. The residue was taken up in a mixture of Et_2O (50 mL) and 1 M $NaHSO_4$ (50 mL). The ethereal fraction was washed with H_2O (3 × 20 mL), brine (2 × 10 mL), dried and concentrated under reduced pressure to give crude monomethyl 5-iodoisophthalate (0.9 g, 94% crude) as a colorless solid which was used for the next step without purification and characterization. R_f = 0.56, EtOAc:hexane=5:7 (3% AcOH).

Boc_2O (1.92 g, 8.80 mmol) was added to a solution of monomethyl 5-iodoisophthalate (0.9 g, 2.93 mmol) and DMAP (0.18 g, 1.47 mmol) in *t*BuOH (30 mL) and the reaction mixture was stirred for 16 h. Afterwards, the mixture was concentrated under reduced pressure, the residue was taken up in Et_2O (50 mL), washed with 1 M $NaHSO_4$ (3 × 20 mL), H_2O (20 mL), 10% $NaHCO_3$ (3 × 20 mL), H_2O (3 × 20 mL), brine (2 × 10 mL), dried, filtered and concentrated under reduced pressure to give crude *tert*-butyl methyl 5-iodoisophthalate (1.05 g, 99% crude) as a yellow oil which was used for the next step without purification and characterization. R_f = 0.56, EtOAc : hexane = 1:4.

1 M NaOH (3.4 mL) was added to a solution of *tert*-butyl methyl 5-iodoisophthalate (1.05 g, 3.1 mmol) in MeOH (10 mL) and THF (5 mL) the reaction mixture was stirred for 2 h at 50°C. Afterwards, the mixture was concentrated under reduced pressure. The residue was taken up in H_2O (50 mL) and washed with Et_2O (2 × 10 mL). The aqueous fraction was acidified to pH = 1 with solid $NaHSO_4$ and extracted with Et_2O (2 × 50 mL). The ethereal solution was washed with H_2O (3 × 20 mL), brine (2 × 10 mL), dried and concentrated under reduced pressure. The residue was purified by column chromatography [$CHCl_3$(3% AcOH)] to give 8 (0.52 g, 48% over 3 steps) as a colorless solid. R_f = 0.35, $CHCl_3$(3% AcOH). ^{1}H NMR (300 MHz, $CDCl_3$) δ 8.90–10.08 (br, 1H) 8.64 (t, *J* = 1.6 Hz, 1H) 8.58 (t, *J* = 1.6 Hz, 1 H) 8.54 (t, *J* = 1.6 Hz, 1H) 1.62 (s, 9H). ^{13}C NMR (75 MHz, $CDCl_3$) δ 169.8, 143.3, 142.5, 134.3, 131.1, 130.3, 93.4, 82.5, 28.1. MS (ESI): positive mode m/z = 1066.9 ([3M + Na]$^+$), 718.9 ([2M + Na]$^+$), 389.0 ([M + K]$^+$), 371.0 ([M + Na]$^+$), 366 ([M + NH_4]$^+$); MS (ESI): negative mode m/z = 695.0 ([2M – H]$^-$), 347.0 ([M – H]$^-$). ESI HRMS: calcd for $C_{12}H_{13}O_4INa^+$: 370.9751; found: 370.9749; calcd for $C_{12}H_{12}O_4I^-$: 346.9786; found: 346.9787.

tert-Butyl3-[(4-{2-[1-(4-chlorobenzoyl)-5-methoxy-2-methylindol-3-yl]acetamido} butyl) carbamoyl]-5-iodobenzoate (9)

WSC·HCl (0.3 g, 1.58 mmol) was added to an ice-cold solution of 8 (0.5 g, 1.44 mmol) and HONSu (0.18 g, 1.58 mmol) in DMF (5 mL). Thereafter, CH_2Cl_2 was added to the reaction mixture until WSC·HCl completely dissolved. The cooling bath was removed and the mixture was stirred for additional 16 h. The reaction mixture was concentrated under reduced pressure, the residue was taken up in Et_2O (70 mL), washed with H_2O (3 × 20 mL), brine (2 × 10 mL), dried and concentrated under reduced pressure to give crude *tert*-butyl *N*-hydroxysuccinimidyl 5-iodoisophthalate (0.63 g, 64%) as a colorless foam which was used for the next step without purification and characterization. R_f = 0.58, EtOAc:hexane = 5:7.

A solution of 4 (0.66 g, 1.42 mmol) in DMF (5 mL) followed by DIEA (0.5 mL, 0.37 g, 2.85 mmol) was added to a solution of *tert*-butyl *N*-hydroxysuccinimidyl 5-iodoisophthalate (0.63 g, 1.42 mmol) in THF (5 mL) and the reaction mixture was stirred for 2.5 h. Thereafter, the mixture was concentrated under reduced pressure. The residue was dissolved in EtOAc (70 mL), washed with 1 M $NaHSO_4$ (3 × 20 mL), H_2O (20 mL), 10% $NaHCO_3$ (3 × 20 mL), H_2O (3 ×20 mL), brine (2 × 10 mL), dried, filtered and concentrated under reduced pressure. The residue was purified by column chromatography ($CHCl_3$:MeOH = 20:1) to give after recrystallization from EtOAc/Et_2O 9 (0.86 g, 80%) as a yellow solid. R_f = 0.35, $CHCl_3$(3% AcOH). ^{1}H NMR (300 MHz, $CDCl_3$) δ 8.39 (t, *J* = 1.5 Hz, 1H), 8.30–8.33 (m, 1H), 8.27–8.30 (m, 1H), 7.62–7.69 (m, 2H), 7.44–7.51 (m, 2H), 6.91 (d, *J* = 2.6 Hz, 1H), 6.85 (d, *J* = 9.0 Hz, 1H), 6.77 (t, *J* = 5.9 Hz, 1H), 6.69 (dd, *J* = 9.0, 2.6 Hz, 1H), 5.92 (t, *J* = 5.9 Hz, 1H), 3.79 (s, 3H), 3.66 (s, 2H), 3.40 (q, *J* = 6.0 Hz, 2H), 3.26 (q, *J* = 6.0 Hz, 2H), 1.60 (s, 9H), 1.47–1.56 (m, 4H). ^{13}C NMR (75 MHz, $CDCl_3$) δ 170.3, 168.3, 165.2, 163.5, 156.2, 140.8, 140.0, 139.6, 136.42, 136.36, 133.9, 133.5, 131.2, 131.0, 130.3, 129.2, 126.8, 115.1, 112.8, 112.2, 101.2, 93.8, 82.3, 55.9, 39.7, 39.0, 32.2, 28.1, 27.0, 26.5 (×2), 13.3. MS (ESI): positive mode m/z = 780.10 ([M + Na]$^+$); MS (ESI): negative mode m/z = 756.2 ([M – H]$^-$). ESI HRMS: calcd for $C_{35}H_{37}N_3O_6ClINa^+$: 780.1308; found: 780.1294; calcd for $C_{35}H_{36}N_3O_6ClI^-$: 756.1343; found: 756.1344.

3-[(4-{2-[1-(4-chlorobenzoyl)-5-methoxy-2-methylindol-3-yl]acetamido}butyl) carbamoyl]-5-iodobenzoic acid (7)

TFA (2 mL) was added to a suspension of 9 (0.12 g, 0.16 mmol) in CH_2Cl_2/TIS/H_2O (1 mL/0.15 mL/0.15 mL) and the reaction mixture was stirred for 15 min. The reaction mixture was concentrated under reduced pressure. The residue was triturated with Et_2O and the resulting amorphous solid was recrystallized from MeOH/Et_2O to give 7 (94 mg, 85%) as a yellow solid. R_f = 0.23, $CHCl_3$:MeOH=35:1 (3% AcOH). ^{1}H NMR (500 MHz, $CDCl_3$) δ 13.0–13.80 (br, 1H), 8.69 (t, *J* = 5.5 Hz, 1H), 8.37–8.43 (m, 2H), 8.33 (t, *J* = 1.4 Hz, 1H), 7.98 (t, *J* = 5.5 Hz, 1H), 7.57–7.71 (m, 4H), 7.11 (d, *J* = 2.5 Hz, 1H), 6.94 (d, *J* = 9.0 Hz, 1H), 6.69 (dd, *J* = 9.0, 2.5 Hz, 1H), 3.74 (s, 3H), 3.49 (s, 2H), 3.26 (q, *J* = 6.3 Hz, 2H), 3.09 (q, *J* = 6.3 Hz, 2H), 2.22 (s, 3H), 1.55 (m, 4H). ^{13}C NMR (126 MHz, $CDCl_3$) δ 169.1, 167.8, 165.4, 163.7, 155.5, 139.7, 139.5, 137.5, 136.7, 135.0, 134.2, 132.9, 131.0, 130.8, 130.2, 128.9, 127.4, 114.43, 114.37, 111.2, 101.8, 94.4, 55.4, 38.4, 31.2, 26.6, 26.3, 13.3. MS

(ESI): positive mode m/z = 724.0 ([M + Na]$^+$); MS (ESI): negative mode m/z = 700.1 ([M – H]$^-$). ESI HRMS: calcd for $C_{31}H_{29}N_3O_6ClNa^+$: 724.0682; found: 724.0675; calcd for $C_{31}H_{28}N_3O_6ClI^-$: 700.0717; found: 700.0714.

Indomethacin conjugate 10 [Ind-NH-(CH_2)$_4$-NH-3-$SnBu_3$-Bz]

Labeling precursor for 5. Bu_6Sn_2 (0.26 mL, 0.30 g, 0.52 mmol) followed by $Pd(PPh_3)Cl_2$ (10 mg, 14.3 µmol) were added to a suspension of 5 (0.28 g, 0.44 mmol) in 1,4-dioxane (5 mL) and the reaction mixture was stirred for 16 h at 85°C. The resulting black solution was cooled down to ambient temperature and concentrated under reduced pressure: The residue was thoroughly triturated with pentane (5 × 10 mL) and quickly washed with Et_2O (2 × 5 mL). The solid residue was taken up into CH_2Cl_2 (20 mL), the resulting suspension was filtered through Celite® and the solution was concentrated under reduced pressure. The residue was purified by column chromatography (MeOH:$CHCl_3$ = 1:30; silica gel with 0.1% CaO) to give 10 (0.12 g, 36%) as a colorless solid.

R_f = 0.29, $CHCl_3$:MeOH = 30:1. ^{1}H NMR (600 MHz, $CDCl_3$) δ 7.87 (s, 1H), 7.64 (d, J = 8.4 Hz, 2H), 7.59 (dd, J = 25.6, 7.5 Hz, 2H), 7.46 (d, J = 8.4 Hz, 2H), 7.35 (t, J = 7.5 Hz, 1H), 6.91 (d, J = 2.3 Hz, 1H), 6.85 (d, J = 9.0 Hz, 1H), 6.67 (dd, J = 9.0, 2.3 Hz, 1H), 6.39 (t, J = 5.6 Hz, 1H), 5.91 (t, J = 5.6 Hz, 1H), 3.77 (s, 3H), 3.64 (s, 2H), 3.40 (q, J = 6.3 Hz, 2H), 3.26 (q, J = 6.3 Hz, 2H), 2.38 (s, 3H), 1.61–1.43 (m, 10H), 1.32 (dt, J = 14.7, 7.4 Hz, 6H), 1.11–1.04 (m, 6H), 0.87 (t, J = 7.3 Hz, 9H).^{13}C NMR (150.9 MHz, $CDCl_3$) δ 170.1, 168.3, 168.2, 156.2, 142.9, 139.53, 139.50, 136.4, 134.8, 133.8, 133.6, 131.2, 130.9, 130.3, 129.2, 127.7, 126.2, 115.1, 112.9, 112.1, 101.0, 55.8, 39.5, 39.2, 32.3, 29.0, 27.3, 26.9, 26.8, 13.6, 13.3, 9.6. ESI HRMS: calcd for $C_{42}H_{56}N_3O_4ClSnNa^+$: 844.2876; found: 844.2875; correct isotopic pattern.

Indomethacin conjugate 11 [Ind-NH-(CH_2)$_4$-NH-5-$SnBu_3$-Nic]

Labeling precursor for 6. Bu_6Sn_2 (0.16 mL, 0.19 g, 0.33 mmol) followed by 1,4-dioxane (3 mL) were added to a solution of 6 (0.2 g, 0.31 mmol) and $Pd(PPh_3)Cl_2$ (10 mg, 14.3 µmol) in DMF (2 mL) and the reaction mixture was stirred for 16 h at 90°C. The resulting black suspension was cooled down to ambient temperature, filtered through Celite® and the resulting solution was concentrated under reduced pressure. The residue was recrystallized from Et_2O/pentane and the crude product was purified by column chromatography (MeOH:$CHCl_3$ = 1:20; silica gel with 0.1% CaO) to give 11 (0.15 g, 60%) as a colorless solid.

R_f = 0.20, $CHCl_3$:MeOH = 20:1. ^{1}H NMR (300 MHz, $CDCl_3$) δ 8.89 (d, J = 2.2 Hz, 1H), 8.68 (d, J = 1.5 Hz, 1H), 8.24 (dd, J = 2.2, 1.5 Hz, 1H), 7.38–7.74 (m, 4 H), 6.78–7.03 (m, 3 H), 6.62–6.73 (m, 1 H), 5.90 (t, J = 6.0 Hz, 1H), 3.79 (s, 3H), 3.65 (s, 2H), 3.39–3.52 (m, 2H), 3.22–3.35 (m, 2H), 2.39 (s, 3H), 1.54 (m, 8H), 1.33 (dq, J = 14.6, 7.3 Hz, 6H), 1.05–1.21 (m, 6H), 0.72–0.99 (m, 9H).^{13}C NMR (75 MHz, $CDCl_3$) δ 170.2, 168.3, 166.3, 158.0, 156.2, 146.9, 143.4, 139.6, 137.4, 136.4, 133.5, 131.2, 131.0, 130.3, 129.9, 129.2, 115.1, 112.8, 112.1, 101.1, 55.8, 39.7, 39.0, 32.2, 28.9, 27.3, 26.3 (×2), 13.6, 13.2, 9.7. MS (ESI): positive mode m/z = 845.2 ([M + Na]$^+$), 823.3 ([M + H]$^+$); MS (ESI): negative mode m/z = 821.3 ([M – H]$^-$). ESI HRMS: calcd for $C_{41}H_{55}N_4O_4ClSnNa^+$: 845.2828; found: 844.2821; calcd for $C_{41}H_{56}N_4O_4ClSn^+$: 823.3008; found: 823.2995; calcd for $C_{41}H_{56}N_4O_4ClSn^-$: 821.2863; found: 821.2854; correct isotopic pattern.

tert-Butyl3-[(4-{2-[1-(4-chlorobenzoyl)-5-methoxy-2-methylindol-3-yl]acetamido} butyl)carbamoyl]-5-(tributylstannyl)benzoate (12): Labeling precursor for 7. Bu_6Sn_2 (0.16 mL, 0.185 g, 0.32 mmol) was added to a suspension of 9 (0.21 g, 0.28 mmol) and $Pd(PPh_3)Cl_2$ (10 mg, 14.3 µmol) in 1,4-dioxane (2 mL) and the reaction mixture was stirred at 80°C for 16 h. Afterwards, the mixture was concentrated under reduced pressure, the residue was triturated with hexane and the resulting amorphous solid was filtered off. The crude product was purified by column chromatography (EtOAc:hexane = 1:2.5; silica gel with 0.1% CaO) followed by recrystallization from Et_2O/hexane to give 12 (0.14 g, 55%) as an amorphous colorless solid. R_f = 0.37, EtOAc:hexane = 1:2.5. ^{1}H NMR [300 MHz, $(CD_3)_2CO$] δ 8.33 (t, J = 1.8 Hz, 1H), 8.19–8.26 (m, 2H), 7.99 (t, J = 5.3 Hz, 1H), 7.67–7.76 (m, 2H), 7.55–7.61 (m, 2H), 7.22 (t, J = 5.3 Hz, 1H), 7.12 (d, J = 2.5 Hz, 1H), 6.99 (d, J = 9.0 Hz, 1H), 6.67 (dd, J = 9.0, 2.5 Hz, 1H), 3.77 (s, 3H), 3.58 (s, 2H), 3.36 (q, J = 6.4 Hz, 2H), 3.21 (q, J = 6.4 Hz, 2H), 2.30 (s, 3H), 1.51–1.71 (m, 10H), 1.58 (s, 9H), 1.34 (dq, J = 14.7, 7.3 Hz, 6H), 1.12–1.20 (m, 6H), 0.87 (t, J = 7.3 Hz, 9H). ^{13}C NMR [75 MHz, $(CD_3)_2CO$] δ 170.3, 168.9, 167.1, 166.0, 157.2, 143.6, 140.2, 139.9, 139.2, 136.5, 135.8, 135.6, 132.24, 132.19, 132.1, 131.9, 130.0, 128.6, 115.7, 115.1, 112.5, 102.5, 81.7, 56.0, 40.3, 39.8, 32.7, 29.9, 28.4, 28.1, 28.0, 27.7, 14.0, 13.8, 10.3. MS (ESI): positive mode m/z = 942.3 ([M + Na]$^+$); MS (ESI): negative mode m/z = 966.4 ([M + HCO_2H – H]$^-$), 920.4 ([M – H]$^-$). ESI HRMS: calcd for $C_{47}H_{64}N_3O_6ClSnNa^+$: 942.3393; found: 942.3393; correct isotopic pattern.

Radiochemistry

Ind-NH-(CH_2)$_4$-NH-3-[I-125]I-Bz ([I-125]5)

Preparation of [I-125]5 was conducted in a 1.5 mL micro centrifuge tube at room temperature (24°C). 5 µL 15 mM tributylstannyl precursor 10 in methylene chloride (stored at + 4°C) were added and evaporated to dryness. The residue was re-dissolved in 20 µL acetic acid and 15 µL n.c.a. [I-125]iodide in 0.1 M NaOH aq (30–100 MBq)

were added. Labeling reaction was started with addition of 15 µL 2.36 mM CAT in MeCN. It was stopped after 10 min by addition of 10 µL 0.1 M Na2S2O3 and diluted by adding 40 µL 40% EtOH aq. The crude mixture was injected into a 500 µL HPLC loop and a HPLC separation was performed. Conditons: column: Multospher C2; flow: 1 mL/min; eluent: 45% EtOH/55% PBS; elution times: thiosulfate (2.4 min), [I-125]iodide (2.7 min), [I-125]5 (5.2 min); precursor elutes with 80% MeCN aq after ca. 3 min. The collected product fraction (4.5 to 5.8 min, 1.3 mL, 50 to 150 MBq [I-125]5) was directly used for cell uptake experiments. Effective RCY (radiochemical yield) was 80 ± 5%. Quality control: conditions: column: Chromolith; flow: 3 mL/min; gradient elution; eluent-A: MeCN, eluent-B: 0.15 M NaCl aq, gradient: 0.5 à 8 min: 5 à 80% A; injection volume: 20 µL; retention times: thiosulfate (0.2 min), [I-125]iodide (0.2 min), [I-125]5 (5.8 min); precursor 10 (9.1 min). RCP was > 99%, specific activity at EOS: 50 ± 10 GBq/µmol.

Ind-NH-$(CH_2)_4$-NH-5-[I-124/125]I-Nic ([I-124/125]6)

Preparation of [I-124/125]6 was conducted similarly to [I-125]5. However, addition of non-radioactive I-127 was mandatory. Preparation of [I-124/125]6 was conducted in a 1.5 mL micro centrifuge tube at room temperature (24°C). 15 µL HOAc, 5 µL 15 mM tributylstannyl precursor 11 in HOAc (freshly prepared) and 1 µL 1 mM KI followed by 15 µL n.c.a. [I-124/125] iodide (100 – 600 MBq) in 0.1 M NaOH aq were added, resulting in specific activities of 44–70 GBq/µmol for I-125 and 92–400 GBq/µmol for I-124. Labeling reaction was started with addition of 15 µL 2.36 mM CAT in MeCN. It was stopped after 10 min by addition of 40 µL 0.1 Na2S2O3 in 66% MeOH aq and diluted by adding 40 µL HPLC eluent B. The entire crude mixture was injected into a 500 µL HPLC loop, the syringe flushed with additional 40 µL eluent B and a HPLC separation using analytical conditions was performed. Conditions: column: Chromolith; flow: 3 mL/min; gradient elution: conditions identical to quality control. Effective RCY of this step was 75 ± 15%. Collected product fraction (4.7 to 5.4 min, 2.1 mL, 75 to 450 MBq [I-124/125]6) was diluted with 8 mL water, extracted by the C18 cartridge, washed with 1 mL water, and eluted with 1 mL MeCN. The eluate was evaporated at 70°C in a gas stream just until dryness (ca. 15 min), redissolved in 25 µL ethanol, and diluted by 500 µL isotonic saline to be ready for cell uptake and µ-PET animal experiments. RCY was 65 ± 10% for the cartridge step and 45 ± 15 % for the overall RCY. Quality control: conditions: column: Chromolith; flow: 3 mL/min; gradient elution; eluent-A: MeCN, eluent-B: 0.15 M NaCl aq with 1% HOAc, gradient: 0.5 à 8 min: 5 à 80% A; injection volume: 20 µL; retention times: thiosulfate (0.2 min), [I-125]iodide (0.2 min), [I-125]6 (4.8 min); precursor 11 (7.7 min). RCP of the product was > 95%, specific activity at EOS: 43 ± 7 GBq/µmol and 3.5 ± 0.5 GBq/µmol for [I-124]6 and [I-125]6, respectively.

Ind-NH-$(CH_2)_4$-NH-5-[I-125]I-Iphth ([I-125]7)

Preparation of [I-125]7 was conducted similarly to [I-125]5. The synthesis of [I-125]7 was conducted in a 1.5 mL sealed v-shaped GC sample vial at room temperature (24°C). First, 5 µL 15 mM tributylstannyl precursor 12 in CH2Cl2 were evaporated in a N2 stream to dryness and redissolved in 20 µL HOAc. 15 µL [I-125]iodide in 0.1 M NaOH aq were added (30–100 MBq). Labeling reaction started with addition of 25 µL 0.236 mM CAT in MeCN. After 20 min of reaction the tBu ester was cleaved by addition of 200 µL TFA and a reaction at 70°C for 12 min. The crude mixture was diluted with 100 µL water, injected into a 500 µL HPLC loop, and a HPLC separation was performed. Conditions: column: Multospher C2; flow: 1 mL/min; isocratic elution: conditions 40% EtOH aq. Effective RCY of this step was 60 ± 15%. Collected product fraction (3.0 to 4.5 min, 1.5 mL, 15 to 50 MBq [I-125]7) was ready for cell uptake experiments. Overal RCY was 50 ± 15%. Quality control: conditions: column: Chromolith; flow: 3 mL/min; gradient elution; eluent-A: MeCN, eluent-B: 0.15 M NaCl aq with 1% HOAc, gradient: 0.5 → 8 min: 5 → 80% A; injection volume: 20 µL; retention times: [I-125]iodide (0.2 min), TFA (0.2 min), cleaved precursor (4.6 min), [I-125]7 (5.1 min); precursor (10 min). RCP of the product was > 95%, specific activity at EOS: 0.2 ± 0.1 GBq/µmol as determined by the hydrolyzed precursor which could not be separated properly from the product using the Multospher C2 column.

REFERENCES

1. Uddin MJ, Crews BC, Blobaum AL, Kingsley PJ, Gorden DL, McIntyre JO, Matrisian LM, Subbaramaiah K, Dannenberg AJ, Piston DW, Marnett LJ. Selective Visualization of Cyclooxygenase-2 in Inflammation and Cancer by Targeted Fluorescent Imaging Agents. Cancer Res. 2010; 70:3618–27.
2. Seprodi J, Coy DH, Vilchez-Martinez JA, Pedroza E, A.V. S. Branched-chain analogues of luteinizing hormone-releasing hormone. J Med Chem. 1978; 21:276–80.
3. Uddin J, Crews BC, Blobaum AL, Kingsley PJ, Ghebreselasie K, Saleh SS, et al. Synthesis and evaluation of [123I]-Indomethacin derivatives as COX-2 targeted imaging agents. J Label Compd Radiopharm. 2009; 52:387–93.
4. Majumdar S, Burgman M, Haselton N, Grinnell S, Ocampo J, Pasternak AR, Pasternak GW. Generation of novel radiolabeled opiates through site-selective iodination. Bioorg Med Chem Lett. 2011; 21:4001–4.
5. Graf R. Über die N-Methylolamide einiger Pyridin- und Chinolinmonocarbonsäuren. J Prakt Chem. 1933; 138:244–58.
